# An experimental heat wave changes immune defense and life history traits in a freshwater snail

**DOI:** 10.1002/ece3.874

**Published:** 2013-12-10

**Authors:** Katja Leicht, Jukka Jokela, Otto Seppälä

**Affiliations:** 1 Eawag, Swiss Federal Institute of Aquatic Science and Technology8600, Dübendorf, Switzerland; 2 Department of Biological and Environmental Science, University of JyväskyläPO Box 35, Jyväskylä, 40014, Finland; 3 ETH Zürich, Institute of Integrative Biology (IBZ)8092, Zürich, Switzerland

**Keywords:** Global warming, immune function, life history traits, *Lymnaea stagnalis*, resource allocation.

## Abstract

The predicted increase in frequency and severity of heat waves due to climate change is expected to alter disease dynamics by reducing hosts' ability to resist infections. This could take place via two different mechanisms: (1) through general reduction in hosts' performance under harsh environmental conditions and/or (2) through altered resource allocation that reduces expression of defense traits in order to maintain other traits. We tested these alternative hypotheses by measuring the effect of an experimental heat wave (25 vs. 15°C) on the constitutive level of immune defense (hemocyte concentration, phenoloxidase [PO]-like activity, antibacterial activity of hemolymph), and life history traits (growth and number of oviposited eggs) of the great pond snail *Lymnaea stagnalis*. We also manipulated the exposure time to high temperature (1, 3, 5, 7, 9, or 11 days). We found that if the exposure to high temperature lasted <1 week, immune function was not affected. However, when the exposure lasted longer than that, the level of snails' immune function (hemocyte concentration and PO-like activity) was reduced. Snails' growth and reproduction increased within the first week of exposure to high temperature. However, longer exposures did not lead to a further increase in cumulative reproductive output. Our results show that short experimental heat waves do not alter immune function but lead to plastic responses that increase snails' growth and reproduction. Thus, although the relative expression of traits changes, short experimental heat waves do not impair snails' defenses. Negative effects on performance get pronounced when the heat waves are prolonged suggesting that high performance cannot be maintained over long time periods. This ultimately reduces the levels of defense traits.

## Introduction

Temperature is one of the most important environmental factors affecting performance and life histories of organisms (Angilletta 2009). In particular, poikilothermic organisms depend strongly on ambient temperature and respond physiologically to temperature variation. While daily and seasonal changes in temperature are largely predictable and can be counteracted with acclimation processes (Kingsolver and Huey 1998; Sørensen and Loeschcke 2002), the unpredictable occurrence of extreme temperatures can seriously impair organisms' overall performance (Wegner et al. 2008; Roth et al. 2010; Roux et al. 2010). This is because rapidly acting protection mechanisms that increase thermal tolerance are energetically costly (see Feder and Hofmann 1999; Somero 2002 for review), and when temperature becomes too extreme, enzymatic function, membrane structure, and oxygen supply can be impaired (Pörtner et al. 2005). Therefore, extreme weather events are expected to cause severe perturbations in organismal function.

Such unpredictable extreme weather conditions (e.g., heat waves) are expected to become more frequent, more intense, and last for longer time periods in the near future due to global climate change (Easterling et al. 2000; Meehl and Tebaldi 2004; Diffenbaugh et al. 2005). As such events are known to modify many life history traits including organisms' growth rate, longevity, and reproduction (Baldwin and Dingle 1986; Partridge et al. 1995; Parmesan 2006; Adamo and Lovett 2011), climate change may have wide implications on natural populations and ecosystems (see Sarmento et al. 2010; Walther 2010 for review, Montoya and Raffaelli 2010). In addition, extreme weather events may alter ecological interactions including predator–prey (see Parmesan 2006; McCluney et al. 2012 for review) and host–parasite interactions (see Harvell et al. 2002; Hance et al. 2007 for review, Mangal et al. 2008). For example, extreme temperature can increase parasite virulence (Poulin 2006; Wegner et al. 2008) and reduce host immune defense (Roth et al. 2010; Karl et al. 2011; Seppälä and Jokela 2011; Murdock et al. 2012).

Reduced immune function has been demonstrated as a response to several harsh environmental factors and can be due to two different mechanisms. First, host performance could be reduced overall, which would be seen as a decrease across a broad range of host traits (McNamara and Buchanan 2005; Marshall and Sinclair 2010). We call this a general reduction hypothesis. Second, natural selection could favor plastic responses that optimize trait expression by compromising defense traits in order to maintain other traits to maximize fitness under suboptimal conditions (Hoffmann and Hercus 2000; Stearns 2000; Stefano et al. 2002; Adamo and Lovett 2011). We call this an altered resource allocation hypothesis. Additionally, the relative role of the above mechanisms in altering hosts' performance may depend on severity (e.g., duration) of extreme conditions. Thus, in order to understand the mechanisms underlying impaired resistance and their relative importance, it is necessary to couple the observed changes in hosts' defense mechanisms and/or susceptibility to infections over time with simultaneous responses in other life history traits.

In this study, we tested these alternative hypotheses (general reduction vs. altered resource allocation) by investigating the effects of an experimental heat wave on a freshwater snail, *Lymnaea stagnalis*. In its natural habitats, *L. stagnalis* is predicted to experience increasingly intense heat waves in future (Schär et al. 2004). Furthermore, in this system, exposure to high temperature is known to impair the constitutive level of snails' immune function (Seppälä and Jokela 2011). We measured three immune traits important for snails' innate immune system (hemocyte concentration, phenoloxidase [PO]-like activity, and antibacterial activity of hemolymph). From life history traits, we chose to measure growth and reproductive output as they are strongly connected to fitness and commonly traded off with immune defense in other systems (Nordling et al. 1998; French et al. 2007). We chose 25°C as a high (i.e., heat wave) temperature as it lies above snails' thermal optimum (Vaughn 1953), but occurs intermittently in snails' habitats during hot summers (A. Laurila, 2010, unpubl. data; U. Tobler, 2010, unpubl. data). We used 15°C as a control temperature as it is close to snails' thermal optimum (Vaughn 1953) and common in ponds (A. Laurila, 2010, unpubl. data; U. Tobler, 2010, unpubl. data). We exposed snails to these temperatures for up to 11 days as the present-day observations from Western Europe show that heat waves last on average 8.40 days (Meehl and Tebaldi 2004). We predict that if the first hypothesis (general reduction) applies, we would observe an immediate decrease in both immune and life history traits. Alternatively, if the second hypothesis (altered resource allocation) applies, we predict that snails reduce expression of immune traits in order to maintain reproductive life history traits. Furthermore, if the relative role of the above mechanisms depends on the length of exposure to high temperature, we expect general negative effects to become more pronounced over time.

## Material and Methods

### Experimental animals

*Lymnaea stagnalis* is a hermaphroditic snail that is common in ponds and lakes throughout the holarctic region. In its natural habitats, *L. stagnalis* is an important host for various parasites including several highly virulent trematode species (Väyrynen et al. 2000; Faltýnková et al. 2007) that castrate the snails and increase their mortality (Karvonen et al. 2004). Snails used in this study came from a laboratory stock population (F_2_ generation) originating from a pond in Zürich, Switzerland (47°22′N, 8°34′E). The population was maintained in water tanks (temperature ranging from 12 to 20°C, but being close to 15°C for most of the year) for 1 year before the experiment. All snails used in the experiment were at reproductive age. One week prior to the experiment, experimental snails were randomly chosen from the population and placed individually in plastic cups filled with 2 dL of aged tap water. Snails were maintained at 15 ± 1°C and fed ad libitum with spinach to acclimate them to the experimental conditions. During that time, half of the water in the cups was changed daily.

### Experimental design

Experimental snails were maintained as described earlier, and each of them was randomly assigned into one of the two temperature (15 ± 1 and 25 ± 1°C) and to one of the six exposure time treatments (1, 3, 5, 7, 9, and 11 days; 30 snails per treatment combination. Initial size (i.e., shell length) of the snails (range: 24–42 mm) did not differ between the temperature and exposure time treatments (analysis of variance [ANOVA]: *P* > 0.05 for both). At the beginning of the experiment, snails exposed to high temperature were slowly warmed up to 25°C (over 10 h). At the end of the experiment, snails' shell length was measured to the nearest 0.1 mm, and immune traits and reproduction were measured as described below. Snails' energy reserves were measured and analyzed as described in the Appendix S1. A total of twelve snails died during the experiment, and not all traits could be measured from 36 individuals. As the mortality of snails during the experiment was generally low (3.8%), survival could not be used as a response variable to examine the effects of experimental treatments. Therefore, these snails were excluded from the data.

### Effects of temperature and exposure time on immune traits

Hemocyte concentration, PO-like activity, and antibacterial activity of snail hemolymph were measured to determine the functional response of snails' immune system to different temperatures. Measuring snail immune function rather than the susceptibility to some specific parasite species was chosen because examining several immune parameters gives a broad estimate of host defenses, whereas studies focusing on a certain host–parasite interaction are necessarily specific to the particular parasite species being used. Such studies can also be confounded by direct effects of temperature on parasite transmission stages (Haas 1994; Pechenik and Fried 1995; McCarthy 1999). Hemocytes, through phagocytosis, constitute the main part of the cellular immune response (van der Knaap et al. 1993). Hemocytes also synthesize prophenoloxidase (pro-PO), which in the active form PO catalyzes oxidative defense against parasites (Cerenius and Söderhäll 2004). Note that the amount of active PO in snail hemolymph, not the amount of zymogenic pro-PO was measured in this study. As a further component of the innate immune defense, antibacterial peptides are used against microorganisms (Imler and Bulet 2005). The measured immune parameters are important components of immune defense in invertebrates including mollusks, although they have also other functions (Butt et al. 2006; Hellio et al. 2007; e.g., Mitta et al. 2000; Seppälä and Leicht 2013). Also in *L. stagnalis*, these immune parameters are known to be involved in defense against infections as they respond to immune elicitors (Seppälä and Leicht 2013).

To collect hemolymph samples for the immune measurements, each snail was removed from its cup at the end of its temperature treatment (i.e., after 1, 3, 5, 7, 9, or 11 days of exposure), blot dried, and its foot was gently tapped to induce the expulsion of blood (Sminia 1981). Hemolymph samples to measure PO-like activity (10 *μ*L of hemolymph mixed with 100 *μ*L of phosphate-buffered saline [PBS, pH 7.4]) and level of antibacterial activity (100 *μ*L of pure hemolymph) were snap frozen in liquid nitrogen and stored at −80°C for later analysis (see below). Fresh hemolymph was used to measure its hemocyte concentration (cells per *μ*L). To prevent hemocytes from sticking together, 7 *μ*L of hemolymph was mixed with 7 *μ*L of EDTA solution (Sigma-Aldrich, Steinheim, Germany, 4 mg per ml in H_2_O) before counting the cells using a Neubauer hemocytometer (Blau Brand, Wertheim, Germany).

At a later date, PO-like activity was measured with a spectrophotometer using a microtiter plate reader (Infinite 200; Tecan, Salzburg, Austria). Samples were thawed on ice and centrifuged at 4000 *g* for 15 min, after which the supernatant was collected. Each well of a microtiter plate was filled with 140 *μ*L of cold distilled water, 20 *μ*L of cold PBS, and 40 *μ*L of a sample. After this, 20 *μ*L of l-Dopa (4 mg/mL in H_2_O) was added into each well. In the following reaction, the enzyme PO oxidizes l-Dopa to dopachrom, which leads to an increase in the optical density (OD) of the solution. In five wells per plate, the hemolymph sample was replaced with distilled water to control for nonenzymatic oxidation of l-Dopa. The OD was measured at 480 nm immediately after l-Dopa was added and again after a 6-h incubation period at 30°C (instrument measurement range 0–4 OD with 0 being completely transparent and 4 being nontransparent). During that period, the increase in OD over time is linear (O. Seppälä, 2007, unpubl. data). PO-like activity was calculated by subtracting the OD directly after adding l-Dopa from the OD after 6 h. Mean change in the OD of the controls was subtracted from all measurements, and change in OD was calculated in milliunits.

To measure the antibacterial activity of snail hemolymph, 50 *μ*L of hemolymph was mixed with 200 *μ*L of a solution containing lyophilized *Escherichia coli* cells (Sigma-Aldrich; 0.35 mg bacteria cells per ml sodium phosphate buffer, pH 6) in wells of microtiter plates at 20°C. In the reaction, antibacterial enzymes destroy *E. coli* cells. This leads to a decrease in OD of the solution over time. In five wells per plate, the hemolymph sample was replaced with distilled water to control for changes in OD not caused by antibacterial activity. OD was measured at 450 nm immediately after mixing the hemolymph and bacteria and again after 30 min using a microtiter plate reader (Infinite 200; Tecan). During that period, the decrease in OD over time is linear (O. Seppälä, 2007, unpubl. data). The decrease in OD was calculated by subtracting OD after 30 min from OD at the first measurement. Mean change in controls was subtracted from all measurements, and change in OD was calculated in milliunits.

All immune parameters were measured twice from a subsample of snails (*N* = 29 or *N* = 30 depending on the parameter) to estimate repeatability (*R*) of the measurements. Repeatability describes the proportion of variance in a character occurring among rather than within individuals. We calculated it from variance components derived from an ANOVA where individual was used as a factor (Krebs 1989). Repeatability of all parameters was high (hemocyte concentration: *R* = 0.992, *F*_29,30_ = 62.773, *P* < 0.001; PO-like activity: *R* = 0.884, *F*_29,30_ = 3.712, *P* < 0.001; antibacterial activity: *R* = 0.931, *F*_28,29_ = 6.716, *P* < 0.001).

### Effects of temperature and exposure time on reproduction

Reproduction of snails during the experiment was measured at the end of their temperature treatment (i.e., after 1, 3, 5, 7, 9, or 11 days of exposure) using photographs taken from all the egg clutches oviposited by the snails. The images were analyzed using ImageJ (ImageJ 1.42q, Wayne Rasband; National Institute of Health, Bethesda, MD) to estimate the number of eggs in the clutches. The area containing nine to twelve eggs in each clutch was measured as well as the area of the clutch, and an estimate of the total number of eggs in the clutch was calculated. The number of eggs in the clutches oviposited by each snail was then summed up to get a measure of its reproductive output.

### Statistical analyses

To examine the effects of temperature and exposure time on snails' immune function, variation in snails' immune defense traits was first analyzed using a multivariate analysis of variance (MANOVA, with Pillai's trace test statistic for unequal sample sizes). In the analysis, hemocyte concentration (ln transformed), PO-like activity (ln transformed), and antibacterial activity of snail hemolymph were used as response variables. In the analysis, a model with temperature (15 and 25°C) and exposure time (1, 3, 5, 7, 9, and 11 days) as fixed factors was used. As the MANOVA indicated an effect of temperature on immune function (see Results), separate ANOVAs using a similar model as above were conducted for different immune parameters to investigate whether their responses to temperature differed. When statistically significant interactions between temperature and exposure time (indicating dependence of the effect of temperature on the length of exposure) were observed in ANOVAs, differences between temperature treatments at each different exposure times were examined with planned contrasts to identify which time points were responsible for the significant interaction term.

The effect of temperature on snails' growth was examined using an analysis of covariance (ANCOVA) with snails' shell length at the end of the experiment as a response variable, temperature and exposure time as fixed factors, and snails' shell length at the beginning of the experiment as a covariate.

To estimate the effect of temperature on snails' reproduction, the variation in the proportion of snails that oviposited eggs in the experiment was first analyzed using a generalized linear model where the reproductive status of the snails (oviposited eggs, did not oviposit eggs) was used as a binomial response variable with logit link function. In this model, temperature and exposure time were used as fixed factors. In addition, as temperature affected snails' growth and thus size at the end of the experiment (see Results), and the capability to reproduce can be size dependent, the proportion of snails that reproduced during the study was also analyzed using a separate model in which snails' shell length at the end of the experiment was included as a covariate. This model reveals the size-independent effect of examined factors on snails' reproduction and estimates whether temperature affects reproduction directly or indirectly (i.e., via larger size). After that, the variation in the number of oviposited eggs was analyzed using only those snails that reproduced during the experiment. The total number of eggs oviposited (square root transformed) by the snails was used as a response variable in an ANOVA with a similar model as above. Because of a significant temperature by exposure time interaction in this model (see Results), differences in the total number of oviposited eggs between temperature treatments at different exposure times were tested using planned contrasts. Furthermore, the data were analyzed separately for different temperature treatments to estimate the effect of exposure time on reproduction at each temperature in detail. In these analyses, ANOVAs with exposure time as a fixed factor fulfilled with repeated contrasts that compared egg numbers between consecutive exposure time treatments were used. These contrasts reveal changes in the cumulative number of oviposited eggs over the course of a heat wave. Analyses comparing only differences between temperature treatments at each exposure time are not able to estimate that as only the total number of eggs oviposited during the experiment was measured, and snails may reproduce intermittently during the experiment. Additionally, an ANCOVA using snails' shell length at the end of the experiment as a covariate was performed using the full model to estimate size-independent effect of examined factors on snails' reproduction as above.

To examine whether immune traits, growth, and reproduction traded off with each other at individual level and/or relationships among them depended on experimental treatments, similar models as above were conducted adding one trait at a time as a covariate (including main effects and factor by covariate interactions). No negative relationships among the traits or interactions among factors and covariates were detected (data not shown).

The assumptions of all the above analyses were fulfilled, and they were performed using IBM SPSS Statistics, version 19.0 software (IBM Corp., Armonk, NY).

## Results

### Effects of temperature and exposure time on immune traits

**Figure 1:**
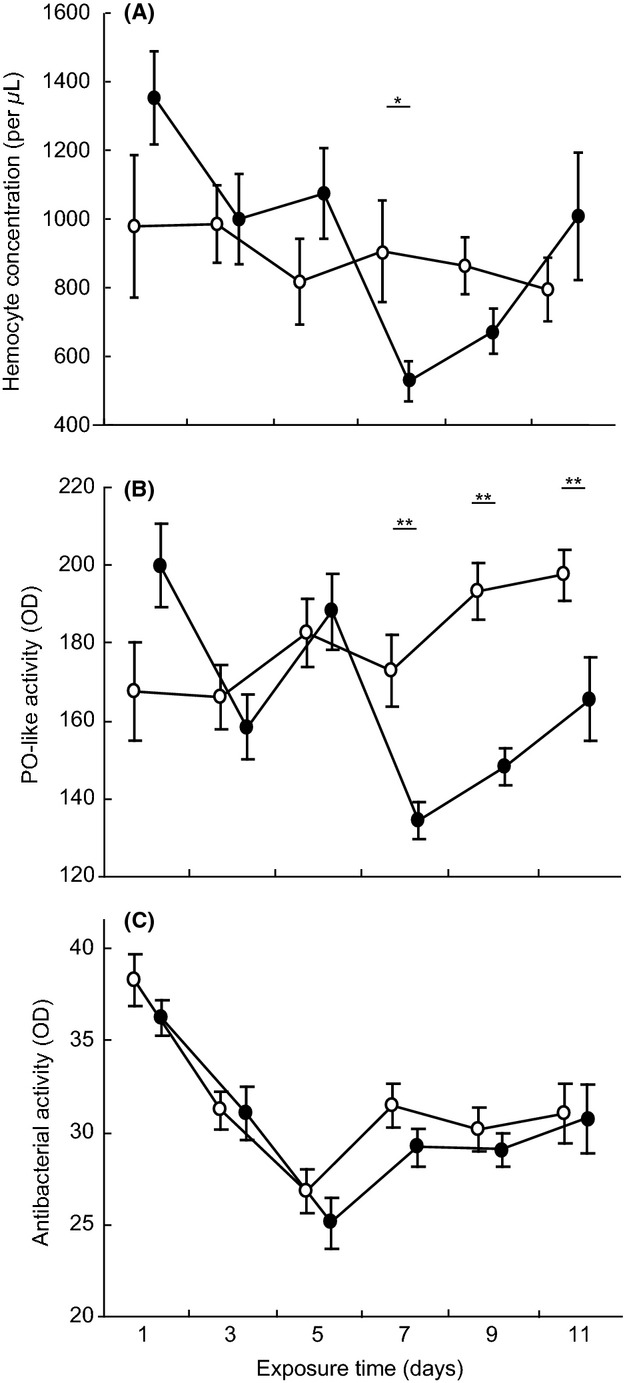
(A) Hemocyte concentration (mean ± SE), (B) phenoloxidase (PO)-like activity (mean ± SE), and (C) antibacterial activity (mean ± SE) of hemolymph for snails exposed to 15°C (open circles) and 25°C (filled circles) for different times (1–11 days).*significance level < 0.05, **significance level < 0.01.

Although the immune function of the snails exposed to 25°C was lower than that of the snails exposed to 15°C (main effect of temperature, MANOVA: Pillai's trace = 0.095, *F*_3,298_ = 10.460, *P* < 0.001), exposure time modified the difference between temperature treatments (temperature × exposure time interaction, MANOVA: Pillai's trace = 0.142, *F*_15,900_ = 2.973, *P* < 0.001). The response to temperature treatment over time was not the same for all the measured immune parameters (Table 1, Fig. 1). When exposed to 25°C, hemocyte concentration of snail hemolymph was reduced in individuals exposed for 7 days (planned contrast: contrast estimate = 0.191, *P* = 0.010; Fig. 1A), but was at the same level as in individuals maintained at 15°C in the subsequent exposure time treatments (planned contrasts: |contrast estimate| ≤0.111, *P* ≥ 0.127 for both; Fig. 1A). PO-like activity was reduced consistently in individuals exposed to high temperature for 7 days or longer (planned contrasts: contrast estimate ≥ 0.086, *P* ≤ 0.005 for all; Fig. 1B). Antibacterial activity of snail hemolymph was not affected by temperature (Table 1, Fig. 1C).

**Table 1:** Analyses of variance (ANOVAs) for the immune parameters (hemocyte concentration, phenoloxidase [PO]-like activity, antibacterial activity of snail hemolymph) by water temperature (15, 25°C) and exposure time (1, 3, 5, 7, 9, and 11 days).

Source	df	MS	*F*	*P*
Hemocyte concentration				
Temperature (*T*)	1	0.034	0.462	0.497
Exposure time (*E*)	5	0.289	3.968	0.002
*T*×*E*	5	0.295	4.044	0.001
Error	300	0.073		
PO-like activity				
Temperature (*T*)	1	0.106	9.92	0.002
Exposure time (*E*)	5	0.054	5.052	<0.001
*T*×*E*	5	0.077	7.223	<0.001
Error	300	0.011		
Antibacterial activity				
Temperature (*T*)	1	118.868	2.795	0.096
Exposure time (*E*)	5	684.786	16.102	<0.001
*T*×*E*	5	10.599	0.249	0.940
Error	300	42.527		

MS, mean squares.

### Effects of temperature and exposure time on growth and reproduction

**Figure 2:**
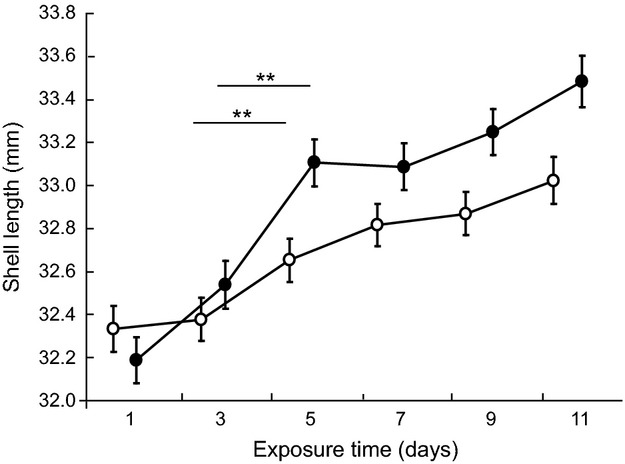
Final size adjusted for initial size (mean ± SE) for snails exposed to 15°C (open circles) and 25°C (filled circles) for different times (1–11 days). **significance level < 0.01

**Figure 3:**
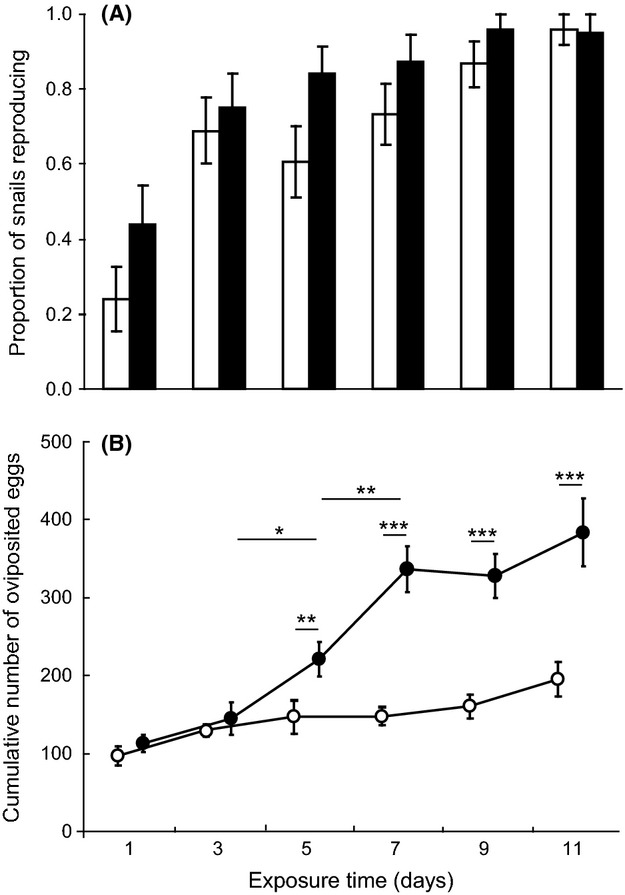
(A) Proportion of snails that oviposited eggs and (B) the number of oviposited eggs for those snails that reproduced in the experiment (mean ± SE) when exposed to 15°C (open bars/circles) and 25°C (filled bars/circles) for different times (1–11 days). *significance level < 0.05, **significance level <0.01, ***significance level < 0.001.

The snails exposed to 25°C grew larger than snails exposed to 15°C (Table 2, Fig. 2). The proportion of snails that oviposited eggs during the experiment was also higher at 25°C (Table 3, Fig. 3A). This effect did not depend on their final size (generalized linear model: Wald χ^2^ = 0.775, *P* = 0.379). For the snails that reproduced, the total number of eggs that were oviposited was higher at 25°C (Table 4, Fig. 3B). The effect of temperature on egg production, however, depended on exposure time (Table 4) so that the number of oviposited eggs by snails exposed to 25°C for 5, 7, 9, and 11 days was higher than the number of eggs oviposited at 15°C (planned contrasts: contrast estimate ≤−3.518, *P* ≤ 0.004 for all). When the total number of eggs was analyzed separately for each temperature treatment, a statistically significant difference in the cumulative number of oviposited eggs among different exposure times was only found in snails exposed to 25°C (ANOVAs: 25°C: *F*_5,110_ = 12.621, *P* < 0.001; 15°C: *F*_5,108_ = 2.130, *P* = 0.067; Fig. 3B). In this treatment, the number of eggs oviposited by the snails differed significantly between individuals exposed for 3 days and for 5 days, and between individuals exposed for 5 days and for 7 days (repeated contrast: contrast estimate ≤−3.007, *P* ≤ 0.025 for both, Fig. 3B). This indicates that the observed increase in egg numbers at 25°C compared with 15°C (see above) was due to higher reproductive rate in short-term exposures to high temperature and that long-term exposures did not lead to a further increase in oviposition. Shell length at the end of the experiment affected the number of eggs oviposited (ANCOVA: *F*_1,217_ = 17.629, *P* < 0.001). However, the effects of other factors remained qualitatively similar in this model when compared with the model without a covariate (Table S1).

**Table 2:** Analysis of covariance (ANCOVA) for snails' shell length at the end of the experiment by water temperature (15, 25°C) and exposure time (1, 3, 5, 7, 9, and 11 days) with initial size as a covariate.

Source	df	MS	*F*	*P*
Temperature (*T*)	1	4.881	16.603	<0.001
Exposure time (*E*)	5	6.830	23.232	<0.001
Initial size	1	2432.713	8274.971	<0.001
*T*×*E*	5	0.622	2.117	0.063
Error	299	0.294		

**Table 3:** Generalized linear model for reproductive status (oviposited eggs, did not oviposit eggs) of snails by water temperature (15, 25°C) and exposure time (1, 3, 5, 7, 9, and 11 days).

	Wald χ^2^	df	*P*
Temperature (*T*)	3.965	1	0.046
Exposure time (*E*)	46.613	5	<0.001
*T*×*E*	1.802	5	0.876

**Table 4:** Analysis of variance (ANOVA) for the total number of oviposited eggs by water temperature (15, 25°C) and exposure time (1, 3, 5, 7, 9, and 11 days).

Source	df	MS	*F*	*P*
Temperature (*T*)	1	801.273	59.528	<0.001
Exposure time (*E*)	5	168.752	12.537	<0.001
*T*×*E*	5	54.898	4.078	0.001
Error	218	13.460		

## Discussion

Extreme weather conditions such as summer heat waves are predicted to become more frequent in the future due to global climate change (Meehl and Tebaldi 2004). This is suggested to have considerable impacts on natural populations and communities, for example, by changing species' ranges, phenology, and interactions among them (see Parmesan 2006; Walther 2010 for review). Here, we show that exposure to high ambient temperature has direct implications for immune defense and life history traits in a freshwater snail *L*. *stagnalis* and that the overall phenotypic response depends on the duration of the experimental heat wave. When exposing snails either to 15°C or 25°C for a maximum of 11 days, we found that short experimental heat waves led to a plastic response that did not alter snails' immune function but increased their growth and reproduction. When the experimental heat wave was longer than a week, the negative effects of high temperature became pronounced. snails' immune function decreased considerably, and reproductive output did not show a further increase compared with short experimental heat waves. This suggests that snails were not able to maintain high performance under increased temperature over a long time period. Thus, only long-term exposures to high temperature can reduce snails' immune defense and predispose them to parasite infections.

The observed response in short experimental heat waves that increased the relative expression of growth and reproduction but did not alter immune defense traits could be explained by adaptive phenotypic plasticity where the optimal resource allocation among traits changed in order to maximize fitness under altered environmental conditions. According to life history theory, optimal resource allocation among traits that are important for self-maintenance (e.g., immune defense) and reproduction depends on the reproductive value of an individual (Fisher 1930). The reproductive value is composed of the current reproductive value and the age-specific shape of the residual reproductive value function and captures the trade-off in resource allocation between maintenance and current reproduction (Fisher 1930; Williams 1966). As exposure to high temperature can increase organisms' metabolic rate and this way fasten aging (Philipp and Abele 2010), this can lead to a shift in the reproductive value, and hence alter allocation of resources among traits. However, contrary to our expectation, immune defense was not reduced at high temperature to maintain other traits, but reproduction and growth were increased while immune function was not affected. This is likely to be due to general physiological changes caused by high temperature that increase performance. According to the van ‘t Hoff and Bredig (1900) principle, an increase of 10°C should enhance the speed of biochemical processes two to fourfold. In fact, the effect of temperature on metabolic rate is demonstrated in many ectothermic species (e.g., Iguchi and Ikeda 1995; Person-Le Ruyet et al. 2004), and this can also explain the increased performance of snails exposed to short experimental heat waves. However, assuming that high temperature was generally beneficial for the physiology, one would also expect an increase in the level of constitutive immune defense traits (Angilletta 2009; Karl et al. 2011). One would especially expect this because higher temperature enhances the growth of potentially pathogenic microorganisms in water (White et al. 1991) that may further activate immune function.

A potential explanation for the negative effects of long experimental heat waves is that decrease in snails' performance reflects the delayed physiological costs of their high growth and reproduction that is induced by short-term response to increased temperature. In our experiment, these costs are unlikely to be due to reduced physiological condition of snails as they were all fed ad libitum, and both lipid and glycogen content of their foot tissue did not differ between the treatment groups (see supplement). Therefore, downregulation of the immune system and reproduction may follow from the demand that physiologically challenging conditions pose on necessary repair and protection mechanisms of tissues when exposure to high temperature continues for a longer period of time. For example, investment in production of heat-shock proteins (Hsp70) is known to increase with increasing temperature (Hofmann and Somero 1995). Additionally, exposure to high temperature may lead to a higher requirement for micronutrients (e.g., zinc, copper, vitamins) (see Askew 1995 for review, Chen et al. 2003) as they are involved in response and tolerance mechanisms (Hänsch and Mendel 2009). Hence, the risk of running out of essential micronutrients can increase with the time organisms are exposed to challenging conditions, and the resulting under-supply may limit performance. In this study, both of these potential explanations are possible and cannot be distinguished. Furthermore, they are probably not mutually exclusive. As both explanations are resource dependent, long experimental heat waves would probably have had a stronger effect if snails were kept under restricted food supply (Moret and Schmid-Hempel 2000; Adamo et al. 2012).

Interestingly, temperature did not have a similar effect on all measured immune parameters in our experiment. While hemocyte concentration and PO-like activity of snail hemolymph were reduced at high temperature, antibacterial activity did not differ between the treatments. The reason for this is unknown, but it is possible that different immune traits respond differently to environmental variation as they are produced through different pathways. For example, the immune traits examined in this study are also known to differ in their responses to starvation of snails (Seppälä and Jokela 2010). It is also possible that antibacterial activity is a more important component of snails' immune defense than hemocyte concentration and PO-like activity and therefore maintained at the same level also under harsh environmental conditions. This could be because different immune parameters have various functions in the snails' immune system. Hemocytes are involved in phagocytosis and encapsulation (as well as in many other functions of the hemolymph) (Sminia et al. 1973; Sminia 1981; van der Knaap et al. 1993), PO is a part of oxidative defenses (Söderhäll and Cerenius 1998; Loker 2010), and antibacterial proteins are specialized against microbial infections (Hancock and Scott 2000). Thus, because of the increased growth rate of microorganisms under high temperatures (White et al. 1991), snails may aim to maintain antibacterial activity of their hemolymph at a high level although reducing other immune traits. Furthermore, it is possible that different immune traits have different thermal optima and therefore vary in their responses to high temperature (Murdock et al. 2012). However, the exact reasons for the differences in the responses of different immune parameters to temperature variation as well as its consequences for snails' defense against different types of parasites remain to be investigated.

Potential ecological consequences entailing these results are manifold. First, the change in reproductive pattern may change snails' population dynamics as well as interactions within and among species (e.g., competition, predation; see Walther 2010 for review). However, the effects of the enhanced reproduction in short-term exposures to high temperature have to be interpreted with caution as we do not know whether temperature affects the quality of the produced offspring (Gilchrist and Huey 2001). Second, snails may become more susceptible to parasites, which can have severe negative impacts on host populations as has been shown in other systems (Massad and Forattini 1998; Pounds et al. 2006). Furthermore, if the above responses of snails to high temperature show genetic variation, this could even alter host–parasite coevolution by changing the effects of host and parasite genetics in determining the outcomes of host–parasite interactions as described in other systems (Blanford et al. 2003; Thomas and Blanford 2003; Mitchell et al. 2005).

In conclusion, short experimental heat waves led to a plastic response that changed the relative expression of fitness related traits by increasing growth and reproduction. This, however, did not impair snails' immune defense. When exposed to long experimental heat waves, negative effects on snails' performance became pronounced as snails' immune function was reduced and reproductive output did not further increase compared with short-term exposures. The fact that snails could not maintain high performance over prolonged experimental heat waves possibly indicates longer term costs of investment in physiological repair and protection mechanisms. Thus, our study demonstrates that more frequent and longer heat waves, as predicted under global climate change scenarios, can alter the expression of life history traits of organisms in natural populations and subject them to parasites by changing the levels of host defenses.

## Supporting Information

**Appendix S1.** Measurement and analyses of energy reserves

**Table S1.** Analysis of covariance (ANCOVA) for the total number of oviposited eggs.
