# Supplementary material for: An experimental heat wave changes immune defense and life history traits in a freshwater snail
Source: Ecol Evol. 2013 Dec 10;3(15):4861–71. doi: 10.1002/ece3.874 (PMC3892353; doi:10.1002/ece3.874)
Supplement: Supplementary file 1 [file ece30003-4861-SD1.pdf]

Appendix S1

Measurement and analyses of energy reserves

Lipid and glycogen content of tissue reflects the energy reserves the snails have available for physiological functions. From a subsample of 81 snails (four to eight individuals per temperature by exposure time combination) tissue samples were taken from the foot (Josse & Van Elk 1986) to measure the lipid and glycogen contents. Samples were snap frozen in liquid nitrogen, and stored at -80°C for later analysis.

To measure the lipid content, tissue samples were dried at 60°C for 48 h. After that, their dry mass was measured to the nearest 0.01mg (balance: Sartorius R200D, precision 0.01 mg). Then each sample was placed in a glass vial and covered with ether. After 24 h, samples were washed with fresh ether to remove all lipids, dried at 60°C for 48 h, and weighted again. The amount of lipids in the tissue was calculated by subtracting the weight after lipids were removed from the weight before lipids were removed.

Glycogen content was measured from 10 mg of tissue. Samples were homogenized in200 µl of cold water. Homogenates were boiled for 5 min to inactivate enzymes. Boiled samples were centrifuged at 13000 rpm for 5 min. Glycogen analyses were done using the EnzyChrom Glycogen Assay Kit (BioAssay Systems, Hayward, CA, USA). OD was measured on 10 µl and glycogen concentrations were determined using a standard curve.

Variation in energy reserves (lipid and glycogen content of snail feet) was analysed with a MANOVA using temperature and exposure time as fixed factors. Lipid content was arcsinesquareroot transformed and glycogen content was squareroot transformed to fulfil the assumptions of MANOVA.

Results of energy reserves

Energy reserves of the snails (i.e. lipid and glycogen content in foot tissue) were not affected by temperature (MANOVA: main effect of temperature: Pillai's trace = 0.043, $F_{2,\,68} = 1.509$, $p = 0.228$; temperature by exposure time interaction: Pillai's trace = 0.186, $F_{10,\,138} = 1.418$, $p = 0.179$). Snails' foot tissue contained on average 0.018 mg lipid/mg dry weight (SE ± 0.061) and 108.528 µg/ml glycogen (SE ± 7.652).

References

Josse, J. & Van Elk, R. (1986) *Trichobilharzia ocellata*: Physiological characterization of giant growth, glycogen depletion, and absence of reproductive activity in the intermediate snail host, *Lymnaea stagnalis*. *Experimental Parasitology,* **62,** 1-13.
