# Supplementary material for: An experimental heat wave changes immune defense and life history traits in a freshwater snail
Source: Ecol Evol. 2013 Dec 10;3(15):4861–71. doi: 10.1002/ece3.874 (PMC3892353; doi:10.1002/ece3.874)
Supplement: Supplementary file 2 [file ece30003-4861-SD2.pdf]

Table S1: Analysis of covariance (ANCOVA) for the total number of oviposited eggs by water temperature (15°C, 25°C) and exposure time (1, 3, 5, 7, 9, 11 days) using final size as covariate.

source	df	MS	*F*	*p*
temperature (T)	1	1945.746	62.001	<0.000
exposure time (E)	5	800.178	25.498	<0.000
T × E	5	70.793	2.256	0.049
final size	1	346.268	11.034	0.001
error	299	31.382		
